# Complementary horse-assisted therapy for substance use disorders: a randomized controlled trial

**DOI:** 10.1186/s13722-020-0183-z

**Published:** 2020-02-04

**Authors:** Francesca Gatti, Espen Walderhaug, Ann Kern-Godal, Jeanette Lysell, Espen Ajo Arnevik

**Affiliations:** Department of Addiction Treatment, Oslo University Hospital HF, P.O 4959, 0424 Nydalen, Oslo Norway

**Keywords:** Randomized controlled trial, Addiction, Substance use disorder, SUD, Comorbidities, Dropout, Equine-facilitated psychotherapy, Horse-assisted therapy

## Abstract

**Background:**

Treatment completion is the greatest challenge for the treatment of substance use disorders (SUDs). A previous investigation showed that complementary horse-assisted therapy (cHAT) was associated with higher retention in treatment and completion than standard treatment alone. This randomized controlled trial further explored the benefits of cHAT for patients with SUDs.

**Methods:**

Fifty patients in residential SUD treatment at the Department of Addiction Treatment, Oslo University Hospital, were randomly allocated to either cHAT (cHAT group) or treatment as usual alone (TAU-only group). The primary end-point was treatment completion. Secondary end-points were dropout, transfer to another treatment, and time in treatment.

**Results:**

The multinomial logistic regression analysis found no statistically significant association between intervention (cHAT) and treatment outcome (completion, dropout, transferred) among the 37 participants who were ultimately recruited to the study. Some unforeseen challenges were encountered in the study: a high number of subjects transferred to another treatment, variable attendance at cHAT sessions, and long temporary exits. Nevertheless, 44% of participants in the cHAT group completed their treatment, compared with 32% in the TAU-only group; this observation encourages further investigation in a larger sample.

**Conclusions:**

Though no association was identified between cHAT and treatment retention or completion, our study may have been underpowered. Further work in a larger clinical population is needed; observational studies with repeated measures may also be useful for investigating whether cHAT increases retention in treatment or rates of completion, two important factors for successful SUD treatment.

*Trial registration* The trial was registered and approved on 14 October 2011 by the Regional Committee for Medical and Health Research Ethics with registration number 2011/1642 and registered at ClinicalTrials.gov on 21 February 2013 with registration number NCT01795755

## Background

Motivating patients to remain actively engaged and to complete their treatment is a long-standing and well-recognized challenge in substance use disorder (SUD) treatment [1]. Retention in treatment improves the prognosis for SUD patients [2–4] and their survival [5]. Active engagement in and completion of treatment are associated with better patient post-treatment follow-up [2, 6, 7] and successful treatment outcomes [3, 4, 8, 9].

The majority of SUD patients have comorbid mental disorders [1, 2] of a heterogeneous nature [10]. Mood and anxiety disorders are most commonly reported [2, 11]. The prognosis for SUD patients with one or more comorbid conditions is generally poorer, with higher rates of treatment dropout and relapse [2]. Longer residential treatment, post-discharge follow-up and outpatient services have been found to be effective, but the risk of substance use disorder relapse remains high for many years [2].

Despite the wide diversity in treatment methods [12], several studies have now shown that patient failure to complete therapy often exceeds 50% [4, 8, 13, 14]. Treatment factors, including time in treatment, have been found to predict treatment outcome [3, 13].

Treatment retention and effectiveness have also been linked to common relational factors, such as empathy and alliance [15].

There is a continuous effort to find new treatment modalities that motivate patients to remain for sufficient time in treatment to enable beneficial changes in morbidity [3, 13, 15, 16]. One innovative strategy is animal-assisted therapy [17–21]. Horse (or equine)-assisted/facilitated (psycho) therapy, for which we use the acronym HAT, is an innovative complementary approach to psychotherapy that actively involves horses or other equines in the therapeutic process [19]. HAT presents a unique opportunity to work within a three-way client–horse–therapist treatment relationship in a contextual setting that differs from the usual therapy clinic [19, 20, 22–24]. The inherent characteristics of the horse, such as size, strength, warmth, body language and herd behaviour can be used with therapeutic benefit in work with clients. For instance, learning herd-based and co-operative behaviour from the horse and experiencing new forms of behaviour and feelings are some of the basic aspects employed as psychotherapeutic tools [19, 23, 25, 26]. The horse can be introduced to the client as a metaphor to (1) explain the horse’s behaviour (what is the horse running away from?), (2) discuss props or tools (what does the halter mean to the horse, and what is your halter in life?), (3) help the client relate to life lessons learned (co-operation, what does it mean that you all dismounted to get the horse over the obstacle?) and (4) inferring lessons about coping (when we work through our obstacles, we succeed) [27]. Associative learning is one of the modalities by which horses learn to respond to a stimulus from those in their surroundings (other horses, predators and the environment). The same stimulus provokes the same response; this also applies to the horse–human interaction, and in a therapeutic setting, such predictability in a horse response to a request (e.g. performing an exercise) by the client can be presented as congruent communication. Horses, as prey animals, are particularly good at judging situations, mostly in relation to what may present a threat, but they lack the form of prejudice that humans have. In a therapeutic setting, horses are often perceived as non-judgemental, facilitating the human–horse connection. The horse’s congruency and its non-judgemental and motivational responses are valuable for building self-esteem, confidence, efficacy and mastery [23, 25, 26, 28–30]. The congruence of a horse’s responses to a human request or approach, for example, can be a useful and positive way for the client to see or become more aware of his/her own behaviour and lack of congruence [22, 25]. Social interaction with the horse can shed light on human interactions and their meanings, and on possibilities for behavioural change, helping to restore a concept of relationships based on trust and attachment [19, 22, 25, 31]. During sessions, the horse will respond naturally to environmental factors (for example, the proximity of other horses or a sudden loud noise). Similarly, the horse will react to the physical and emotional state of the patient (for example, a request lacking focus or clarity is unlikely to produce the desired movement from the horse, and an aggressive request may meet with resistance). The therapist leading the process can both read and influence the horse and can provide reflective feedback to the patient on the relationship, reactions and responses between the horse and the patient [19].

Depression [32–34], trauma and anxiety [25, 26, 33–35], eating disorders [36], aggression [37], poor motivation and low self-esteem [25, 26] are among the most commonly cited psychological conditions associated with therapy with horses. These conditions are also common elements of addiction comorbidity [38–40]. In a recently published randomized controlled trial (RCT) study, Nurenberg and colleagues compared equine and canine forms of animal-assisted therapy with standard treatments for 90 hospitalized psychiatric patients to determine the effects on violence. They reported that HAT was significantly associated with reduced aggression in hospitalized patients; no effect was observed in either the group involved with dogs or the group undergoing standard therapy alone [37].

Possible explanations for HAT participants’ remaining in and completing treatment include therapeutic alliance, the environment, physical activity and staff influence. Gender, motivation, engagement and comorbidity are also frequently mentioned in both the HAT and SUD literature as important. However, detailed empirical studies with evidence for these claims are largely missing from both the SUD [13] and HAT [41, 42] literature.

Since 2011, our group has undertaken a series of parallel research projects to investigate the effects of integrating HAT into the standard treatment of patients with SUDs and comorbidities [19, 43, 44]. We designed a non-randomized intention-to-treat study enrolling 108 patients voluntarily upon referral by their treating clinician and performed univariate and multivariate analyses to compare those receiving treatment as usual (TAU; n = 43) with those receiving HAT in addition to TAU (HAT; n = 65) [19]. The results from this preliminary study showed that HAT is a promising complementary therapy associated with higher retention in treatment and completion than standard psychotherapeutic treatment alone: (1) more HAT participants completed treatment (56.9 vs. 14%, P < 0.001), (2) remained in treatment for longer (means of 141 vs. 70 days, P < 0.001) and (3) had a greater chance of completing their treatment than those not given the HAT treatment (odds ratio (OR) 8.4, 95% confidence interval (CI) 2.7–26.4, P < 0.001) [19]. In a parallel study, we investigated the patients’ perspectives on complementary HAT in their therapeutic process and identified the nature of the *relationship*, their *emotional engagement* and their *sense of mastery* as the main aspects perceived by the patients to be motivational during therapy [44].

To our knowledge, no RCT has evaluated the efficacy of complementary HAT for patients with SUDs. Prompted by the positive results of our previous study, we performed an RCT to assess whether the beneficial effect of integrating HAT into a standard therapeutic programme for patients with SUDs could be replicated with a more rigorous study design. We expected that a higher proportion of participants in HAT complementary to the standard treatment (cHAT) programme would complete treatment as planned compared with participants who received the standard therapeutic programme—i.e., TAU-only—alone. As secondary end-points, we expected the cHAT group to have more days in treatment, a lower rate of dropout and a lower rate of transfer to other residential treatments compared with the TAU-only group.

## Methods

### Patients

Study participants were adult inpatients with a primary diagnosis of mental and behavioural disorders due to psychoactive substance use (ICD-10) recruited from the Department of Addiction Treatment (Youth) at Oslo University Hospital. Enrolment started in January 2013 and continued for 3 years. Patients were referred to the study by their therapist and then randomly allocated.

The randomization process followed a card assignment method with black and red playing cards indicating allocation to the TAU-only control group and the cHAT group, respectively. The randomization was stratified into groups of 10 (5 cHAT + 5 TAU-only in random order) to avoid a situation where a long string of one of the groups would receive or not receive cHAT in a specific time period. This was the only stratification performed. The allocations were recorded in a numerical sequence on a worksheet, and the individual notifications containing the respective allocations were placed in numbered envelopes. The researchers performing the randomization were different from those allocating the patients to groups, and the procedure was performed in accordance with allocation concealment. Patients were informed about the aim of the study and design, including the randomized allocation process, and were then invited to participate in the study. They were advised of their right to withdraw at any time without detriment to their therapy, and only those who returned a signed *Research Consent Form* received the envelope with the allocation sheet indicating the assigned group.

All patients entering the treatment programme after 15 January 2013 were eligible for the study, but patients who had previously attended more than four cHAT sessions (beyond the introductory sessions) were excluded. Previous cHAT was the only exclusion criterion.

### Horses

The horses employed as co-facilitators in the cHAT programme are part of the residential herd of horses of Stallen, the Unit of Horse-Assisted Therapy at Oslo University Hospital. They vary in age, size, gender, breed and temperament but do not undergo specific training because it is their instinctive and responsive behaviour elicited in the horse–human interactions that is the functional aspect of this approach. The only prerequisite for their selection as therapy horses is to be in good physical and psychological health, used to human interaction and comfortable working with people with mental and behavioural disorders. In accordance with horse welfare guidelines, the horses live as a herd in a fenced open area to guarantee their natural social interactions and feeding behaviours. Animal welfare is a priority in managing and employing horses for therapy at the Stallen Unit, and the RCT study fully adhered to Stallen’s policy and to national regulations related to animal welfare (*Mattilsynet*—*the Norwegian Food Safety Authority*).

### Study design

This study employed an intention-to-treat, randomized, parallel-controlled design (RCT). The experimental group participated in horse-assisted therapy in adjunct to standard treatment: complementary HAT (cHAT). The control group was offered only the standard treatment: TAU-only.

### Intervention protocols

#### Treatment as usual (TAU-only group)

The standard TAU treatment, described in detail elsewhere [19], is a person-centred treatment programme comprising individual and group therapy. It is based on a biopsychosocial model with an emphasis on mentalization-based theory and practice [26, 45] and tailored to the individual’s specific problems and treatment goals. The likely duration of treatment is decided with the patient as part of the treatment plan, in accordance with his/her needs. This implies that the therapy varies between patients in terms of goals and duration of treatment. To reduce the risk of dropout from the TAU-only programme, subjects in this group were offered the opportunity to participate in cHAT sessions after the study terminated.

#### Complementary horse-assisted therapy (cHAT group)

The cHAT treatment, described in detail elsewhere [19], is a horse-facilitated psychotherapeutic programme provided as an adjunctive intervention (complementary) to the standard treatment (TAU). The cHAT protocol utilized in this study is based on the clinical cHAT protocol employed at our institution (*The ‘Gaustad Model’ of Horse*-*Assisted Therapy at Oslo University Hospital*—unpublished). The cHAT programme started in the second week of treatment and consisted of 12 structured sessions of cHAT, each of 90-min duration, provided twice a week for a total of 6 consecutive weeks. The program is structured for small groups (maximum four participants per session) and involves a three-way interactive process in which the patient works with the horse on activities planned with the therapist to address agreed goals. Activities are designed to address challenges relevant to SUDs, such as boundary setting, development of trust and control of emotional affect. Observation of the herd can promote discussion of social interaction and relationships and stable duties promote responsibility, routine and reliability. Ground work is used to address issues relating to boundaries and contact, anxiety and trust, communication and connection, mastery (of new skills, the horse and self), body awareness and focus. Mounted work addresses posture, balance and centring, co-ordination, rhythm and regulation, mastering of anxiety and focus. Carriage driving can be used to promote forward thinking and outlook, and with other passengers, it can engender a sense of empowerment, group responsibility and care [19]. The first four sessions were *Introductory sessions* mostly focused on instructing the patients on the cHAT programme, horse behaviour and safety precautions. The following eight sessions were *Therapy sessions* addressing treatment goals and involving, at the discretion of the cHAT therapist and according to patient treatment progression, some or all of the following activities: ground work (such as grooming, leading or setting limits), mounted work (riding in the arena, around the grounds or in the woods), vaulting (gymnastics on horseback) and driving a carriage. Although designed as a standard and structured protocol, owing to individual treatment plans and treatment progression, the number and frequency of cHAT sessions were adapted to individual needs, and as a consequence, the cHAT intervention could vary in both content and length among subjects in the cHAT group. The cHAT sessions were planned and provided by highly qualified psychotherapists specializing in equine-facilitated psychotherapy. The outcome variables were retrieved from the clinical records and stored in the Youth Addiction Treatment Evaluation Project (YATEP) database.

### Measures

In this study, treatment completion was the primary end-point. Dropout, transfer to other treatment, time in treatment and attendance at cHAT sessions variables were also analysed as secondary outcomes (attendance at cHAT sessions was added to the study post hoc). The medical records were reviewed by personnel employed in a separate unit, dedicated to patient referral and analysis (Section referral, analysis and patient allocation, Oslo University Hospital). They were blind to the random treatment allocation and unfamiliar with the project or the project group.

The coding of treatment termination in the participants’ medical records adheres to the Norwegian health authority’s codex (*Norwegian Patient Registry*—*Norwegian Directorate of Health*). In our study, each variable was defined and assessed as follows:

Treatment completion: Completion of treatment according to the treatment plan.

Dropout: This implies that the patient leaves the therapeutic programme before completion. Dropout is treatment termination initiated by the patient against medical advice.

Expulsion: Treatment termination initiated by the treatment institution owing to patient misconduct or aggressive behaviour.

Transfer to other treatment: The patient is transferred from the assigned intervention programme to another residential treatment. The reasons for the transfer may include identification of a more suitable programme at a different institution or a limited hosting capacity of the current institution.

Time in treatment: Number of days in treatment at discharge. In a case of temporary exit, the number of days off treatment was subtracted from the total at discharge.

Attendance at the cHAT programme: The cHAT programme consisted of 12 sessions, and attendance was classified as *Low* if a patient had attended fewer than eight cHAT sessions, or *High* if a patient had attended eight or more cHAT sessions. This measurement was added post hoc.

### Statistical analysis

The power calculations used an estimate of 14.0% completion in TAU-only and 56.9% completion in cHAT. Calculating a significance level of *α* = 5% (*P* < 0.05) and the power to detect a difference in magnitude of 1–*β* = 0.80, indicated a total of 50 subjects. This estimation was based on our previous study [19]. Differences in patients’ baseline characteristics between the two groups were analysed with Fisher’s Exact Test. Differences in continuous variables between groups were tested with Student’s t test, and for categorical data, the Chi squared test was used. A Kaplan–Meier survival curve was employed to determine differences in time (days) to treatment completion between cHAT and TAU-only groups. Subjects were censored if they completed their treatment, while dropout was scored as an event. Differences were estimated using log-rank statistics. Multinomial logistic regression analysis was used to investigate the association between intervention (cHAT vs. TAU-only groups) and treatment completion (completion, dropout, transferred) and to adjust for gender and education as possible confounding factors. The effect was quantified by ORs with their 95% CIs. A significance level of 5% was used. The analysis was performed with IBM SPSS Statistics for Windows, version 23 (Armonk, NY, USA; IBM Corp.). Graphs were created with GraphPad Prism, version 5 (GraphPad Software, Inc.).

## Results

### Study participants’ characteristics

**Fig. 1:**
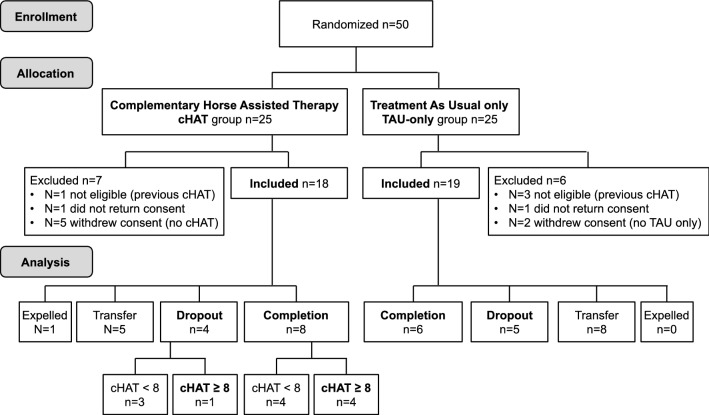
Study flowchart

As the result of the allocation process, 50 patients were assigned to one of the two treatment groups: the intervention, which consisted of horse-assisted therapy complementary to treatment as usual (n = 25, the cHAT group), and the control, which consisted of treatment as usual only (n = 25, the TAU-only group). As shown in the study flow chart (Fig. 1), of 50 patients initially allocated, only 37 (cHAT = 18, TAU-only = 19) could be included in the study, while 13 were excluded for various reasons: four were not eligible (previous participation in cHAT), two did not return a signed consent form and seven withdrew their consent (five from the cHAT group did not want to do cHAT, and two from the TAU-only group did not want to do TAU-only). These subjects were excluded after allocation because their ineligibility was discovered retrospectively.

Despite random allocation, the two groups differed in some demographic and clinical characteristics (Table 1). The TAU-only group had higher male representation (74%), while in the cHAT group, the genders were more equally distributed with slightly higher female representation (56%). In both groups, approximately 80% of the subjects were aged between 20 and 26 years. The level of education, defined as lower education if the participants had 10 years or less of schooling, and higher education if they had more than 10 years of schooling, showed substantial intra- and intergroup disparity, with more subjects with higher education in the cHAT group (61%) than in the TAU-only group (42%). All patients were in-patients with a primary diagnosis of mental and behavioural disorders due to psychoactive substance use (ICD-10). The majority of subjects had multiple drug and psychoactive substance use diagnoses, although there were fewer in the TAU-only group (58%) than in the cHAT group (78%). As for comorbidities (ICD-10), a higher percentage of subjects in the cHAT group presented more severe comorbidity diagnoses, defined as three or more psychiatric disorders (cHAT = 22%, TAU-only = 5%). The most common comorbidities were related to mood disorders (F30–39), neurotic stress/post-traumatic stress disorders (F40–48), personality disorders (F60–69) and behavioural and emotional disorders (F90–98).

**Table 1:** Patients’ demographic and clinical characteristics

Variable	Item	cHAT (N)	cHAT (%)	TAU-only (N)	TAU-only (%)	Total (N)	Total (%)	P value*
Subjects		18	49	19	51	37	100	
Gender	Male	8	44	14	74	22	60	0.099
	Female	10	56	5	26	15	41	
Age (years)	year < 20	1	6	2	11	3	8	0.763
	20 ≤ yea ≤ 26	14	78	15	79	29	78	
	26 < yea ≤ 30	3	17	2	11	5	14	
Schooling (years)	yea ≤ 10	7	39	11	58	18	49	0.330
	yea > 10	11	61	8	42	19	51	
Substances^b^	n = 1	2	11	5	26	12	19	0.541
	n = 2	2	11	3	16	7	14	
	n ≥ 3	14	78	11	58	12	68	
Comorbidities^b^	n = 0	3	17	7	37	10	27	0.349
	n = 1	8	44	7	37	15	41	
	n = 2	3	17	4	21	7	19	
	n ≥ 3	4	22	1	5	5	14	

cHAT patients are those in Horse Assisted Therapy complementary to Treatment As Usual (TAU), and the TAU-only group are patients in Treatment as Usual only. N indicates frequencies, and % indicates proportions as percentages

* P value calculated using Fisher’s Exact Test (* P < 0.05; ** P < 0.01; *** P < 0.001)

^a^Cannabis, alcohol, heroin, amphetamine, benzodiazepine, (GHB) gamma hydroxybutyrate, and cocaine

^b^Mood disorders, neurotic stress/post-traumatic stress disorders, personality disorders and behavioural and emotional disorders

### Treatment outcomes

In this study, we observed three different treatment outcomes: treatment completion (primary), which is the most favourable outcome; dropout, which is an unfavourable outcome; and transfer to another treatment, which is generally considered to be a favourable outcome, although it is characterized by a large variety of individual reasons.

Of 37 subjects ultimately recruited to the study, 14 completed the assigned treatment (38%), 9 dropped out of treatment (24%), 13 were transferred to another residential treatment (35%) and one was expelled (3%). For the two intervention groups, the outcomes were as follows: (1) more subjects in the cHAT group completed their assigned programme (44%) than for the TAU-only group (32%), (2) slightly fewer patients in the cHAT group dropped out of treatment (22%) than with controls (26%) and (3) fewer subjects in the cHAT group were transferred to another treatment (28%) than with controls (42%; Table 2).

**Table 2:** Treatment Outcomes

Variable	Item	cHAT (N)	cHAT (%)	TAU-only (N)	TAU-only (%)	Total (N)	Total (%)	P value*
Subjects		18	49	19	51	37	100	
Treatment outcome	Completion	8	44	6	32	14	38	0.640
	Dropout	4	22	5	26	9	24	
	Transfer	5	28	8	42	13	35	
	Expelled	1	6	0	0	1	3	

Descriptive analysis of outcomes in cHAT and TAU-only groups

* P value calculated using Fisher’s Exact Test (* P < 0.05; ** P < 0.01; *** P < 0.001). cHAT vs. TAU-only

The multinomial logistic regression analysis found no statistically significant association between the patients receiving intervention compared to TAU and dropout relative to completion (OR: 0.60, 95% CI 0.11–3.25, *P*-value: 0.553), nor any statistically significant association between the patients receiving intervention compared to TAU and transfer relative to completion (OR: 0.47, 95% CI 0.10–2.19, P-value: 0.335). The results did not change after adjusting for the confounding effect of gender and employment (Table 3).

**Table 3:** Multinomial logistic regression analysis of treatment outcomes

	P value *Unadjusted*	Odds Ratio	Lower	Upper	P value Adjusted*	O.R.	Lower	Upper
Dropout	0.553	0.600	0.111	3.245	0.471	0.499	0.075	3.302
Transferred	0.335	0.469	0.101	2.185	0.153	0.255	0.039	1.664

cHAT vs. TAU-only; completers are the reference; 95% Confidence Interval

* Adjusted for gender and education

### Time in treatment

**Fig. 2:**
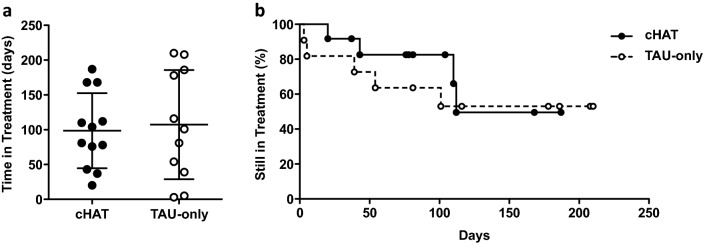
Treatment outcomes. **a** Time in treatment for subjects in cHAT vs. TAU-only. Data are represented as a scatter dot plot, where each dot indicates a subject at the time of discharge (days). The mean with SD is reported. **b** Survival curve representing treatment completion and dropout events for patients in complementary HAT (cHAT) vs. Treatment as Usual only (TAU-only) group, relative to time in treatment (days). Each dot indicates a subject. Treatment completion (censored subject) is indicated by a dot on the horizontal line; dropout (event) is indicated by a dot on a downward step (in the cHAT group, two subjects were censored on day 168)

In this study, we did not observe a statistically significant difference in time in treatment between the two groups (Fig. 2a). The subjects in the cHAT group showed an average time in treatment of 98.7 days (± 5.6 days) and the controls showed an average of 107.4 days (± (23.65 days, *P* = 0.237). Treatment completion and dropout events relative to time in treatment are shown in Fig. 2b (Hazard Ratio 0.733, 95% CI 0.197–2.719, *χ*^2^ 0.709, 1; *P* = 0.400).

### Attendance at the cHAT programme

Although the cHAT protocol prescribed attendance at 12 sessions, only two subjects (11%) attended the full programme, while the rest attended a variable number of sessions, with most of the subjects attending fewer than eight sessions (67%; Table 4). The level of attendance was also characterized by a gender difference: 75% of the male participants vs. 50% of the females attended fewer than eight cHAT sessions (Table 4).

**Table 4:** Attendance at cHAT sessions

Attendance at cHAT	Low (N)	Low (%)	High (N)	High (%)	Tot (N)	Tot (%)
Subjects	12	67	6	33	18	100
Completion	4	50	4	50	8	47
Dropout	3	75	1	25	4	24
Transfer	4	80	1	20	5	29
Male*	3	75	1	25	4	33
Female*	4	50	4	50	8	67

Low attendance corresponds to fewer than eight sessions; high attendance corresponds to eight or more sessions (n = 18)

* Analysis of gender distribution among completion and dropout outcome only (n = 12)

To our knowledge, there is no evidence in the literature on how many sessions a HAT programme should have or the minimal number of sessions to attend to observe a positive effect. Therefore, we conducted a post hoc test to see whether there was an association between the number of cHAT sessions and treatment completion (our primary outcome). The analysis did not show any statistically significant difference between level of attendance (high or low) and treatment completion. We observed that among the subjects who had attended fewer than eight cHAT sessions, 75% had dropped of treatment, and among the subjects who had attended more than eight sessions, 25% had dropped out (OR 3.000, 95% CI 0.211–42.65; *χ*^2^ 0.686, 1; *P* = 0.408). Most of the subjects who were transferred attended fewer than eight sessions (80%), regardless of the duration of treatment (Table 4).

## Discussion

The hypothesis that cHAT would improve treatment retention and completion was based on recently published results from a study by our group on a sample of 108 patients, showing that the subjects in the cHAT programme (n = 65) were more likely to complete their treatment than those attending TAU-only (adj. OR 8.4, 95% CI 2.7–26.4; *P* < 0.001) [19]. The scope of the RCT design was to compensate for the lack of randomization in the previous study, which limited the generalizability of the results to a population potentially more motivated to participate in the cHAT programme. In the current study, we found no association between cHAT and treatment completion and thus were unable to confirm the previously shown statistically significant association of cHAT with treatment completion. We suspect that the reduction in sample size from n = 50 to n = 37 subjects, and the fact that only 2 patients (11%) completed the 12 cHAT sessions, were the main cause of the lack of statistical significance in the results. In our clinical routine, patients can request to participate in the cHAT programme through their therapists or can be referred by them; hence, the findings from this study remain relevant for clinical application.

Using dropout as a standard measurement and comparing it between studies is complicated by the fact that a unique and concordant definition of dropout is lacking [13]. Nevertheless, patient failure to complete therapy, usually defined as dropout, is a commonly used evaluation measure of the SUD treatment process. In our study, 24% of patients dropped out of treatment, which is below the level that we observed in our previous investigation (60%) and the rate often reported in studies of addiction (more than 50%) [4, 8, 13, 14].

In the TAU-only programme 42% of subjects were relocated to another residential treatment, compared to 28% in the cHAT programme, but this was not statistically significant. A previous study found that cHAT is a good strategy for retention in treatment [19], but this was not confirmed in this underpowered RCT study. Neither did we find the cHAT programme to be associated with longer times in treatment, as previously reported [19]. The optimal duration of treatment is debatable, and it depends on both the treatment method and the problems of the individual patient [1], but 90 days is often identified as the minimum period for effective treatment [4, 7, 46]. In our previous investigation [19], we found that subjects participating in the cHAT programme remained in treatment for a significantly longer period than those in the TAU-only group (mean 141 days vs. 70 days) and that they were more likely to remain in treatment for 90 days or more. No association between participation in the cHAT programme and longer time in treatment was observed in the current study, although in both treatment groups the average time in treatment was more than 90 days.

To our knowledge, there is no empirical evidence on the minimum number of cHAT sessions required to achieve a treatment benefit. In our protocol, the first four sessions of HAT were introductory, while the following eight were targeted at the therapeutic goal. In the study, most of the subjects attended fewer than eight sessions, indicating an overall low commitment to the cHAT treatment. We decided to analyse whether there was a specific number of cHAT sessions that would give a better outcome (lower dropout rate) and observed that 75% of subjects who had attended fewer than eight cHAT sessions dropped out of treatment, whereas only 25% of subjects who had attended more than eight sessions did so. Despite the cohort being too small to run a statistical analysis, this observation might indicate a need to provide a minimum of eight cHAT sessions in cHAT programmes for the treatment of patients with SUD. The relevance of the number of cHAT sessions for a certain outcome should be assessed individually (e.g. retention in treatment, treatment completion, symptoms reduction) on a larger study sample, and the programme designed accordingly.

Females in treatment for addiction constitute a marginalized group in SUD treatment, where the gender distribution is normally two-thirds male and one-third female [47, 48]. The finding that in the cHAT group females attended a higher number of cHAT sessions than males suggests further investigation of the hypothesis that the HAT programme is a promising treatment option for SUD females.

### Strengths and limitations

The strength of our study is the use of a randomized parallel-groups controlled design. Unfortunately, the sample size was drastically reduced during enrolment, study and final analysis, compromising the statistical analysis and increasing the risk of a *type II error*. The therapeutic efficacy of the cHAT treatment reported here may be an underestimation. This seems probable considering that our data, at least in relation to treatment completion, showed the same trend as that observed in our previous study where patients in cHAT were more likely to complete their treatment than subjects in the TAU-only group [19].

The RCT design in a study assessing an intervention such as cHAT, where a placebo design for the control group is not feasible, and with a clinical population characterized by high rates of dropout and non-compliance with treatment [49], was more difficult to apply than with the non-random allocation of our previous study. Indeed, several patients withdrew their consent because they were not assigned to the treatment programme in which they wished to participate. Moreover, a high number of subjects (67%) in cHAT may have attended an insufficient number of cHAT sessions (cHAT < 8). Notably, the majority of subjects had a diagnosis of multiple drugs and psychoactive substance use, and at least one of concurrent psychopathology, constituting a clinical population with severe SUDs, and such people have notoriously high rates of dropout and low treatment compliance [49].

Both TAU and cHAT are person-centred treatment programmes tailored to individuals’ specific problems and treatment goals. This implies that the therapy varies between patients in terms of goals and duration of treatment. We did not control for the number of treatment sessions (either for TAU or for cHAT), which is a limitation. Another limitation is the lack of a control group for the complementary activity, for example, dog-assisted therapy or gardening, which would have specifically controlled for the horse contribution. Retrieval of information from the hospital clinical records can potentially be affected by subjective interpretation. This is a limitation in our study, but we controlled for the retrieval of the outcome variables by having this done by personnel blind to the experimental conditions and without any affiliation to the authors or study site.

### Further research

In a recent review of animal-assisted therapy for SUDs, Klemetsen and Lindstrøm [50] have highlighted the lack of studies with a strong methodological design, advocating for the urgency of more randomized and controlled studies. Very few peer-reviewed studies investigate HAT for SUD treatment, and none, to our knowledge, assess retention in treatment and employ an RCT design [18–21].

This study did not find a statistical significant effect of cHAT on SUD treatment completion, but the possibility of a *type II error* cannot be ruled out. Future RCT designs on cHAT with treatment completion as outcome variable should include a bigger study population to increase the statistical power. This would most likely promote more reliable results and clear conclusions on the utility of cHAT on SUD treatment completion.

Conducting a study with an RCT design on HAT in a SUD population is difficult and requires substantial resources. Furthermore, it is reasonable to assume that cHAT is more effective for patients actively seeking this treatment than with those not wanting it.

In the future, we hope to see RCT studies on HAT with sufficient statistical power, using outcome variables with large effect sizes. RCT designs give good causal indications, provided they have sufficient time, funding and statistical power. Future studies should control for the number of cHAT sessions or establish a pre hoc minimum number of sessions. We used treatment completion as the outcome variable, but future research should include additional outcome variables specifically related to HAT. In a qualitative study, the participants reported that HAT facilitated positive attachment, reflective functioning, self-efficacy and emotional regulation [44]. All of these, or other outcome variables theoretically associated with HAT, should be investigated. The research literature on HAT would also benefit from studies using longitudinal prospective cohort designs. This observational design cannot establish causation, but it was easier to implement than RCT. A prospective ‘pre–post’ cohort design with repeated measures, for example, could give important indications of the psychometric properties that seem to change over time with HAT, and for whom.

## Conclusion

The aim of our study was to perform a randomized clinical trial to assess the effect of integrating HAT in a therapeutic programme for patients with SUDs as a means to facilitate a positive treatment outcome. We did not find that participants in the cHAT programme had higher rates of completing treatment than participants who received the standard therapeutic programme. More participants assigned to cHAT completed their treatment (44%) than subjects in standard therapy alone (32%), but this was not statistically significant using an RCT design with N = 37 participants ultimately recruited to the study.

## Supplementary information

**Additional file 1.** Consort flow diagram.

**Additional file 2.** Consort checklist.

## Data Availability

The datasets used and/or analysed during the current study are available from the corresponding author on reasonable request (Additional files 1 and 2).

## Abbreviations

HAT: Horse-assisted therapy
cHAT: Complementary horse-assisted therapy
YATEP: Youth Addiction Treatment Evaluation Project
RCT: Randomized controlled trial
SUD: Substance use disorders
TAU: Treatment as usual
