# Supplementary material for: Complementary horse-assisted therapy for substance use disorders: a randomized controlled trial
Source: Addict Sci Clin Pract. 2020 Feb 4;15:7. doi: 10.1186/s13722-020-0183-z (PMC7001193; doi:10.1186/s13722-020-0183-z)
Supplement: Supplementary file 1 — Additional file 1. Consort flow diagram. [file 13722_2020_183_MOESM1_ESM.doc]

**
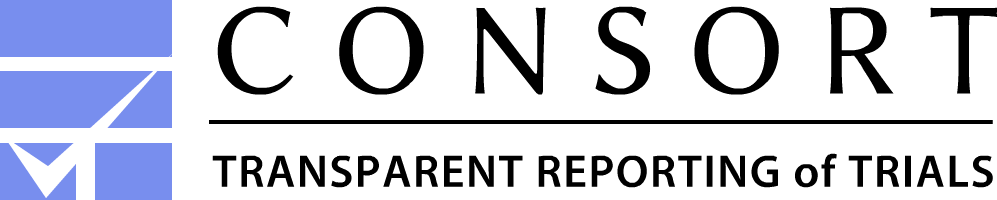
**

**CONSORT 2010 Flow Diagram**

**Allocation**

**Analysis**

**Follow-Up**

**Enrollment**

Assessed for eligibility (n=50)

Excluded (n=13)

♦  Not meeting inclusion criteria (n=4)

♦  Declined to participate (n=7)

♦  Other reasons (n=2)

Analysed (n=12)
♦ Excluded from analysis (n=6): 1 expelled, 5 transferred to other treatment

Lost to follow-up (n=0). Discontinued intervention (n=6): 1 expelled, 5 transferred to other treatment

Allocated to intervention (n=18)

♦ Received allocated intervention (n=18)

♦ Did not receive allocated intervention (give reasons) (n=0)

Lost to follow-up (n=0). Discontinued intervention (n=8): transferred to other treatment

Allocated to intervention (n=19)

♦ Received allocated intervention (n=19)

♦ Did not receive allocated intervention (give reasons) (n=0)

Analysed (n=11)
♦ Excluded from analysis (n=8): transferred to other treatment

Randomized (n=37)
