# Supplementary material for: Complementary horse-assisted therapy for substance use disorders: a randomized controlled trial
Source: Addict Sci Clin Pract. 2020 Feb 4;15:7. doi: 10.1186/s13722-020-0183-z (PMC7001193; doi:10.1186/s13722-020-0183-z)
Supplement: Supplementary file 2 — Additional file 2. Consort checklist. [file 13722_2020_183_MOESM2_ESM.docx]

**Table S1. Patient characteristics and treatment outcome in n = 23 patients.**

| Variable | Item | cHAT (N) | cHAT (%) | TAU-only (N) | TAU-only (%) | Total (N) | Total (%) |
| --- | --- | --- | --- | --- | --- | --- | --- |
| Subjects |  | 12 | 52 | 11 | 48 | 23 | 100 |
| Gender | Male | 4 | 33 | 9 | 82 | 13 | 57 |
|  | Female | 8 | 67 | 2 | 18 | 10 | 43 |
| Age (years) | y < 20 | 1 | 8 | 1 | 9 | 2 | 9 |
|  | 20 ≤ y ≤ 26 | 10 | 83 | 8 | 73 | 18 | 78 |
|  | 26 < y ≤ 30 | 1 | 8 | 2 | 18 | 3 | 13 |
| Schooling (years) | y ≤1 0 | 4 | 33 | 7 | 64 | 18 | 78 |
|  | y > 10 | 8 | 67 | 4 | 36 | 19 | 83 |
| Treatment outcome | Completion | 8 | 67 | 6 | 55 | 14 | 61 |
|  | Dropout | 4 | 33 | 5 | 45 | 9 | 39 |
